# Inefficient DMN Suppression in Schizophrenia Patients with Impaired Cognitive Function but not Patients with Preserved Cognitive Function

**DOI:** 10.1038/srep21657

**Published:** 2016-02-17

**Authors:** Li Zhou, Weidan Pu, Jingjuan Wang, Haihong Liu, Guowei Wu, Chang Liu, Tumbwene E. Mwansisya, Haojuan Tao, Xudong Chen, Xiaojun Huang, Dongsheng Lv, Zhimin Xue, Baoci Shan, Zhening Liu

**Affiliations:** 1 Mental Health Institute, Second Xiangya Hospital, Central South University, Changsha, Hunan, People’s Republic of China; 2 Medical Psychological Institute, Second Xiangya Hospital, Central South University, Changsha, People’s Republic of China; 3 Key Laboratory of Nuclear Analysis Techniques, Institute of High Energy Physics, Chinese Academy of Sciences, Beijing, People’s Republic of China; 4 Mental Heath Centre, Xiangya Hospital, Central South University, Changsha, Hunan, People’s Republic of China; 5 College of Health Sciences, University of Dodoma, P.O. Box 395, Dodoma, Tanzania; 6 Beijing Engineering Research Center of Radio-graphic Techniques and Equipment, Beijing, People’s Republic of China; 7 The China National Clinical Research Center for Mental Health Disorders, National Technology Institute of Psychiatry, Key Laboratory of Psychiatry and Mental Health of Hunan Province, 139 Middle Renmin Road, Changsha, Hunan 410011, People’s Republic of China; 8 State Key Laboratory of Medical Genetics, Central South University, Changsha, Hunan, People’s Republic of China

## Abstract

Previous studies have observed reduced suppression of the default mode network (DMN) during cognitive tasks in schizophrenia, suggesting inefficient DMN suppression is critical for the cognitive deficits of schizophrenia. Cognitive function in schizophrenia patients, however, varies from relatively intact to severely impaired. This study, which compared the DMN suppression patterns between first-episode schizophrenia patients with (SZ-Imp) and without (SZ-Pre) impaired cognitive function, may provide further insight into the role of DMN dysfunction in cognitive deficits of schizophrenia. Independent component analysis (ICA) was applied to resting-state fMRI data to identify the DMN in each subject, and then general linear modeling based on the task-fMRI data was used to examine the different DMN activation patterns between groups. We observed that the SZ-Imp group, but not the SZ-Pre group, showed reduced suppression in the medial prefrontal cortex and posterior cingulated cortex when compared to the healthy controls (HC) group. Moreover, less DMN suppression was associated with poorer task performance in both HC and patient groups. Our findings provide the first direct evidence that disrupted DMN activity only exists in schizophrenia patients with impaired cognitive function, supporting the specific neuro-pathological role of inefficient DMN suppression in cognitive deficits of first-episode schizophrenia.

A large-scale brain network named default mode network (DMN) typically shows increased activation during internal cognitive processing (e.g. self-referential thinking and autobiographic memory), but decreased activation during externally goal-directed cognitive tasks^1^. This default network is composed of a set of brain regions^2^,^3^, including the medial prefrontal cortex (mPFC), posterior cingulate cortex (PCC), precuneus, and lateral/medial temporal lobes. Although the DMN was initially hypothesized to be mainly associated with self-referential and internally directed information processing (such as mind-wandering and day-dreaming)^4^, recent fMRI studies have shifted their focus to its neural contribution to externally goal-directed cognitive functioning^5^,^6^. Converging evidence from fMRI studies based on various cognitive experimental paradigms has shown consistent suppression/deactivation in the DMN areas^1^,^7^. Importantly, greater DMN suppression has been found to be associated with more efficient behavioral performance^8^,^9^, suggesting that the efficient suppression of DMN is critical for optimal cognitive performance in healthy subjects.

Cognitive deficit, one of the most prominent symptoms of schizophrenia, is related to the long-term outcome (i.e., occupational functioning) of this severe mental disorder^10^,^11^. Despite the well-established inverted “U-shaped” relationship between prefrontal activation and behavioral performance in schizophrenia patients^12^,^13^,^14^, recent fMRI studies have also revealed reduced DMN suppression during a broad range of cognitive tasks (such as working memory^15^,^16^, recollection^17^ and selective attention^18^) in this population. One popular notion that has emerged from these studies is that the failure of DMN suppression, reflecting an occupation of cognitive resources by internally directed information processing, leads to an interruption of goal-directed behavior in schizophrenia^5^,^19^,^20^,^21^. However, many studies have failed to detect any reduction of DMN suppression in schizophrenia^22^, and some studies have even shown increased DMN suppression^23^,^24^. The reason for this inconsistency of DMN impairment remains unclear. One possible source of this inconsistency may result from variance in the degree of cognitive impairment in schizophrenia patients^25^, which ranges from relatively intact to severely impaired (DSM-IV)^26^. If the efficient suppression of DMN is important for cognitive functioning, it is reasonable to hypothesize that the schizophrenia patients with preserved cognitive function would present intact DMN suppression during goal-directed cognitive tasks.

To test this hypothesis, the present study recruited twenty-two schizophrenia patients with preserved cognitive function (SZ-Pre), twenty-two patients with impaired cognitive function (SZ-Imp) and twenty-one healthy controls (HC) to compare the DMN suppression patterns among three groups during an n-back working memory task. Meanwhile, to minimize the potential confounding effects of chronic illness course (e.g. hospitalization, social isolation and long-term medication) on cognitive function in schizophrenia, we specifically focused on first-episode schizophrenia patients in this study. Then, we expected that compared to the HC group, the SZ-Imp group but not the SZ-Pre group in first-episode would present reduced DMN suppression and that this DMN dysfunction would be correlated with WM deficit in schizophrenia.

## Methods

### Ethical Statement

All participants gave their written informed consent to participate in our study after detailed description of the risks and benefits. The study was approved by the ethics committee of the Second Xiangya Hospital, Central South University. All the subsequent research analyses were carried out in accordance with the approved guidelines.

### Participants

Using the Structured Clinical Interview for DSM-IV (Diagnostic and Statistical Manual of Mental Disorders, Fourth Edition), Patient version (SCID-I/P)^27^, forty-four first-episode schizophrenic patients were recruited from the Department of Psychiatry, Second Xiangya Hospital of Central South University, Changsha, China. All patients met the following inclusion criteria: 1) at least 9 years of education; 2) a duration of illness less than 18 months with no previous episodes of psychosis; 3) right handedness; 4) no history of chronic neurological disease, substance abuse, electro-convulsive therapy or severe medical disorder. Additional clinical assessments for the patients included the Scale for Assessment of Positive Symptoms (SAPS)^28^ and the Scale for the Assessment of Negative Symptoms (SANS)^29^,^30^ within 1 month prior to the fMRI scanning session. During fMRI scanning, a parametric n-back task was conducted with all participants to evaluate their cognitive function, since working memory (WM) is a well-documented cognitive function that is impaired in schizophrenia^31^. In line with the inclusion criterion of schizophrenia with cognitive deficits in previous studies^13^,^32^,^33^, the patients were divided into SZ-Pre group and SZ-Imp group on the basis of the WM task accuracy (each including twenty-two patients). In accordance with two memory processes (resistance to distraction and information maintenance) and the deletion function included in this WM task^34^, our criterion was based on the 2-back task accuracy (the average of the Target and Non-target accuracy). In particular, each raw 2-back task accuracy score was standardized to a z score (with a mean of 0 and a SD of 1) using the values of HC group as reference. The patients were then classified as impaired if their performance score was more than 1 SD below the normative mean^25^. To validate our grouping results, the patients were further asked outside the scanner to complete the Digital Symbol Substitution Test (DSST) and the Information Subscale of Wechsler Adult Intelligence Scale Chinese Revised (WAIS-CR)^35^,^36^ which measure, respectively, information processing speed and verbal comprehension, two other important cognitive functions. Two-sample *t* test showed that both information processing speed and verbal comprehension differed significantly between the SZ-Imp and SZ-Pre group (*t* = 2.331, *p* = 0.026; *t* = 1.997, *p* = 0.044; respectively) (Table 1), providing robust support for our grouping criterion based on the WM task performance. Importantly, to demonstrate the specific relationship between DMN suppression abnormality and the cognitive deficits in first-episode schizophrenia, the clinical and demographical variables, including SAPS total score (*t* = 0.150, *p* = 0.107), SANS total score (*t* = 0.659, *p* = 0.087), age at illness onset (*t* = 0.519, *p* = 0.607), medication dosage (*t* = 1.160, *p* = 0.254), duration of illness (*t* = 0.519, *p* = 0.607), age (*t* = 0.536, *p* = 0.620), sex (*χ*^*2*^ = 0.519, *p* = 0.176), and years of education (*t* = 0.547, *p* = 0.110) were all matched between the two patient groups (Table 1).

Twenty-one healthy controls matched for sex, age and education were recruited for comparison. The inclusion and exclusion criteria were the same as those for the patients, except that the controls did not meet the DSM-IV criteria for any mental disorders and had no first-degree relatives with a history of any psychiatric disorders.

### MRI protocol

The task-based and resting-state fMRI image data were acquired using a Philips Gyroscan Achieva 3.0 Tesla MRI scanner in the axial direction, using a gradient-echo echo-planar imaging (EPI) sequence: repetition time (TR) = 2000 ms, echo time (TE) = 30 ms, flip angle (FA) = 90°, matrix = 64 × 64, slice thickness = 4 mm, gap = 0 mm, slices = 36, 250 time points. High-resolution T1-weighted images were also acquired with a three-dimensional spoiled gradient echo (SPGR) pulse sequence from the sagittal plane, scanning parameter: TR = 7.5 ms, TE = 3.7 ms, FA = 8°, 180 slices, matrix = 256 × 200, the field of view (FOV) = 240 × 240 mm^2^, and slices were contiguous with slice thickness of 1 mm. Importantly, during the T1-weighted and resting-state images acquisition, all the participants were asked to rest with their eyes closed and try not to think of anything systematically.

### n-back task

As described in our previous studies^35^,^37^, all participants performed a parametric n-back task on NordicNeurolab’s fMRI hardware system for 8 minutes and 16 seconds. Initially, a short fixation period was presented for 4 s before WM task to allow for MRI scanner calibration. Then tasks were presented in a blocked design with four 40 s blocks of the “0-back” task (condition “A”), alternating with four 40 s blocks of the “2-back” task (condition “B”). Each task block was preceded by a task instruction for 2 seconds. A 20 s fixation period, which provided a recovery period for the hemodynamic response between task blocks^38^, was inserted after each task block. The data from these fixation periods were also used as the pre-stimulus baseline (condition “rest”). All stimuli were sequences of white capital letters on a black background, presented centrally (500 ms duration, 1500 ms inter-stimulus interval) in pseudo-random order.

Participants were instructed to respond to every stimulus using a 2-button response box, with one button used to signal targets (35% of the stimuli in each task block) and one used to signal non-targets. To perform the 0-back task, participants were instructed to identify the target letter “X”, and identify all other letters as non-targets. To perform the 2-back task, participants were instructed to press the right button when the current letter matched the one presented two trials back (targets) and the left button when the current letter did not match (non-target)^39^. The task performance, as represented by the reaction time [RT] and accuracy, of each participant was recorded electronically by the computer connected to the fMRI.

### Statistical Analysis

Non-fMRI data were analyzed using the Statistical Package for the Social Sciences (SPSS) software (standard version 20.0). To compare the clinical characteristics between groups, *t-*test or analysis of variance (ANOVA) was used for continuous variables and Chi-square test was used for ratios and proportions. fMRI data were analyzed using the SPM8 package (http://www.fil.ion.ucl.ac.uk/spm) running on MATLAB 2010a (The MathWorks Inc. Sherbon MA, USA).

### Image preprocessing

For each participant, the first ten volumes of the resting-state fMRI data for participants to get used to the circumstances and the first two volumes of the task-based fMRI data for scanner calibration (A short fixation epoch was presented in the screen for 4 s before the first block of WM task at the beginning of the task-fMRI scanning) were removed. The following preprocessing was applied to the remaining 248 volumes of the task-based fMRI data and 240 volumes of the rest-fMRI data: slice time correction and realignment allowing a maximum displacement and/or angular rotation of less than 2 mm/2° in the x, y, and z planes. Of the forty-four patients in this study, three patients with preserved cognitive function and six with impaired cognitive function were excluded due to excessive head movement artefacts (i.e. rotations larger than 2° or translations greater than 2 mm) during fMRI scanning. The remaining nineteen SZ-Pre patients, sixteen SZ-Imp patients and twenty-one healthy participants were included in further analyses, and there were no significant differences in 6 head movement parameters between the three groups (please see Supplemental Table S2). Subsequent preprocessing included six rigid head motion parameters correction, spatial normalization to the Montreal Neurological Institute echo-planar imaging template (each voxel was resampled to 3*3*3 mm^3^), and spatial smoothing with a Gaussian kernel (full width at half-maximum, 8 mm).

### Independent Component Analysis and Identification of DMNs

Spatial independent component analysis (ICA) was conducted on the resting-state fMRI data for all fifty-six participants using the Group ICA of fMRI Toolbox (GIFT) software (Medical Image Analysis Lab, University of New Mexico, Albuquerque, New Mexico; http://icatb.sourceforge.net/). The exact pipeline for ICA has been applied in our prior works^40^ and other studies^41^,^42^,^43^,^44^,^45^. Based on the aggregate dataset from all participants, data dimensionality (number of components) was estimated using the minimum description length criteria tool in GIFT^41^,^44^,^45^, which suggested that 30 is the optimal number of independent components (ICs). The dimensions of the functional data were then reduced using principal component analysis^41^,^42^, followed by an independent component estimation that produced spatial maps and time courses with the infomax algorithm^42^. Estimated ICs at the group level (both spatial maps and time courses) were then back-reconstructed for each participant based on principal components analysis compression and projection^41^,^43^,^44^, yielding subject-specific spatial maps and time courses for each estimated component. This specific back-reconstruction feature of the GIFT algorithm allows analysis of all participants simultaneously as part of a large ICA group matrix^43^. For each IC, the time courses of each component therefore represented a pattern of synchronized brain activity, whose coherency pattern across voxels was represented in the associated spatial map. To display voxels relevant to a particular IC, the intensity values in each map were converted to z values^46^.

**Figure 1:**
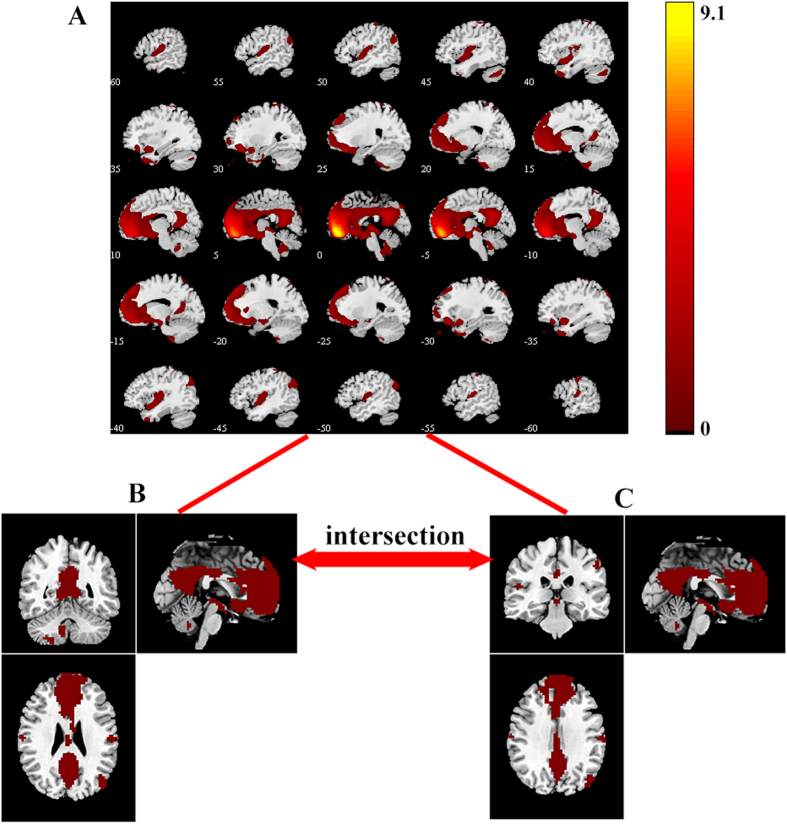
Masks of the default mode network in the HC and SZ groups. (**A**). The DMN was determined by independent component analysis (ICA) in all participants. (**B**). The DMN mask of HC group was identified by one sample *t*-test (n = 21, *p* < 0.05 with FWE correction); (**C**). The DMN mask of SZ group was identified by one sample *t*-test (n = 35, *p* < 0.05 with FWE correction). The DMN masks from the HC and SZ groups were further intersected into one mask for task-induced activation analysis. DMN, default mode network; HC, healthy controls; SZ, schizophrenia. The color bar represents the T values.

To identify the valid default-mode network, a template of the DMN was used to select the best-fit component for each participant. The standard DMN template was from a meta-analytic modeling provided by Angela R. Laird, Ph.D. (Research Imaging Institute, University of Texas Health Science Center, San Antonio, Texas)^47^. After the best-fit component representing the DMN was extracted from each participant, the components were gathered from HC and SZ group (combining the SZ-Imp and SZ-Pre group) separately for a random effect analysis using the one-sample *t* test. The significant threshold was set at *p* < 0.05 with family-wise error (FWE) correction. The DMN masks from the HC and SZ groups were then intersected into one mask for further task-induced activation analysis (Fig. 1).

## Task-fMRI Data Analyses

### First-Level Analyses

Within-subject analyses (i.e., fixed effect) used a block-based general liner model. Each experimental block (2-back, 0-back, and rest) was modeled using a boxcar function convolved with a canonical hemodynamic response function. To correct for head motion, the six realignment parameters were included in the design matrix as regressors of no interest. In previous studies^1^,^48^, DMN activity has been consistently found to be decreased during externally goal-directed cognitive tasks when compared to the resting state, traditionally referred to “DMN suppression or deactivation”. Since we were specifically interested in the role of DMN suppression in cognitive impairments of schizophrenia, contrasts were created for each participant comparing the 2-back task condition versus the ‘rest’ condition. The contrast images for each participant were submitted to a second-level analysis (i.e., random effect) for making inferences at the group level.

### Second-Level Analyses

Within-group effects were tested using one-sample *t* tests with FWE correction (*p* < 0.05) on contrast images (2-back task condition versus ‘rest’ condition) in each group separately. Between-group differences were tested using two-sample *t* tests between three groups (*p* < 0.05 with FWE correction at the cluster-lever, and uncorrected *p* < 0.001 at the voxel-level). The group comparisons were restricted (masked) to the voxels within the DMN mask (Fig. 1). In regions showing between-group differences in activation, the measure of activation response (the first eigenvalue) was extracted from each participant’s data, and the effect size (Cohen’s *d*) for group status on brain activation was calculated. Pearson analysis was used to evaluate the relationship between the DMN activity with group difference and the 2-back task performances, clinical symptoms, duration of illness, and medication dosage.

## Results

### Generic task-induced activation and deactivation in three groups

**Figure 2:**
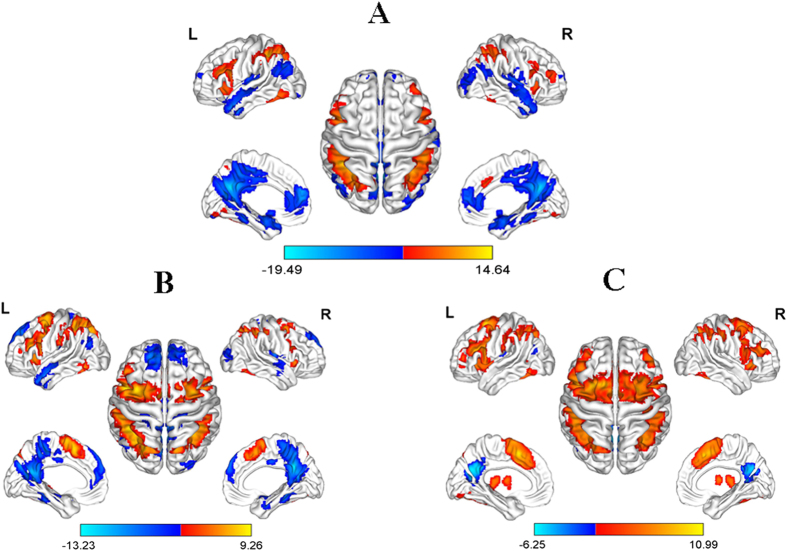
Significant (de)activated regions during 2-back working memory task in three groups (*p* < 0.05 with FWE correction). The red-yellow color represents the activated regions, while the blue color represents the deactivated regions. (**A**) healthy controls; (**B**) schizophrenia patients with preserved cognitive function; (**C**) schizophrenia patients with impaired cognitive function. L, left hemisphere; R, right hemisphere. The color bar represents the T values.

One-sample *t* test with FWE correction showed that in the HC group (Fig. 2), the dorsal prefrontal cortex (*p* = 0.001, Cohen’s *d* = 2.53), parietal cortex (*p* = 8.0754*10^−8^, Cohen’s *d* = 3.14) and cerebellum (*p* = 1.3089*10^−5^, Cohen’s *d* = 2.25) were significantly activated, while the DMN regions including mPFC (*p* = 2.3040*10^−7^, Cohen’s *d* = −2.46), PCC/precuneus (*p* = 4.5158*10^−6^, Cohen’s *d* = −3.31), middle temporal gyrus (*p* = 1.3204*10^−5^, Cohen’s *d* = −2.41) and para-hippocampal gyrus (*p* = 3.5664*10^−6^, Cohen’s *d* = −2.54) were significantly deactivated during the 2-back WM task. In the two patient groups (Fig. 2), despite the similar activations in the WM-related prefrontal and parietal cortex, while the SZ-Pre group shared similarities of DMN deactivation with the HC group, the SZ-Imp group only showed significant DMN deactivation in the PCC (*p*  =  0.048, Cohen’s *d* = −1.32).

### Differences in DMN-suppression among three groups

**Figure 3:**
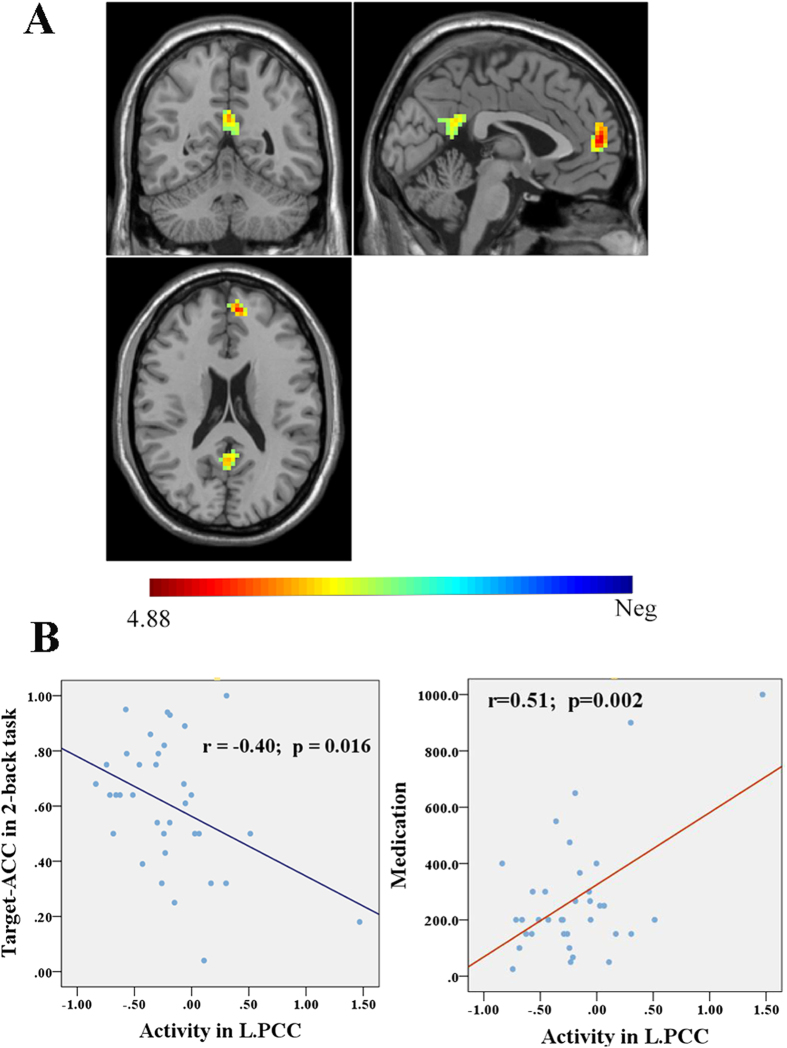
Differences of DMN activity in the 2-back working memory task between healthy controls and schizophrenia patients with impaired cognitive function. (**A**) shows greater activity (decreased suppression) in the left mPFC and PCC in schizophrenia patients with impaired cognitive function compared to health controls (cluster *p* < 0.05 with FWE correction); (**B**) shows the significant correlations of the left PCC activity with Target-accuracy of 2-back task and with medication dosage in patients. mPFC, medial prefrontal cortex; PCC, posterior cingulated cortex. The color bar represents the T values.

Compared to the HC group, the SZ-Pre group did not show any significant differences of brain activity in the DMN regions, while the SZ-Imp group showed reduced deactivation in the left mPFC (MNI = [−6, 54, 9], cluster = 85, *p* = 0.001 with FWE correction, Cohen’s *d* = 1.59) and the left PCC (MNI = [0, −54, 24], cluster = 50, *p* = 0.026 with FWE correction, Cohen’s *d* = 1.38) (Fig. 3). Comparison between the two patient groups showed no significant differences of activity in the DMN regions under the threshold of *p* < 0.05 with FWE correction. But under the uncorrected threshold of *p* < 0.001, the SZ-Imp group showed higher PCC activity compared to the SZ-Pre group (MNI = [−3, 54, 18], cluster = 13, Cohen’s *d* = 1.39).

Additionally, the whole brain analysis showed that the mPFC, PCC, para-hippocampus, temporal pole and angular gyrus in the DMN regions presented reduced deactivation only in the SZ-Imp group compared to the HC group (*p* < 0.001, uncorrected); meanwhile, the regions relevant to cognitive functions (such as anterior insula, anterior cingulated cortex and caudate)^49^ showed increased activation in the SZ-Imp patients relative to the healthy controls (see the Supplementary Materials, Table S1). Particularly, the increased activation in the anterior insula was also found in the SZ-Pre group compared to the HC group.

### Correlation between task-induced DMN-suppression and clinical/behavioral measures

In the patient group, the left PCC activity was negatively correlated with Target-accuracy of 2-back task (r = −0.40, *p* = 0.016) (Fig. 3B), while in the HC group, the left PCC activity was positively correlated with Non-target-RT of 2-back task (r = 0.49, *p* = 0.025). However, no significant correlation was found between the left PCC activity and Non-target-RT of 2-back task in the patient group (r = 0.11, *p* = 0.548) or between the left PCC activity and Target-accuracy of 2-back task in the HC group (r = −0.30, *p* = 0.187). To test whether the relationship of the left PCC activity with WM performances was equal between patients and healthy controls, we performed the Fisher r-to-z transform and hypothesis test, and found that there were no differences in the correlation of the left PCC activity with Target-accuracy (z = 0.39, *p* > 0.1) or Non-target-RT (z = 1.46, *p* > 0.05) between the HC and SZ group.

In addition, medication dosage was positively correlated with the left PCC activity (r = 0.51, *p* = 0.002) in schizophrenia patients (Fig. 3B). No significant correlations were found between brain activity in the DMN regions and the SAPS/SANS scores, age at illness onset or duration of illness in patients.

## Discussion

This study used a task fMRI approach to assess the DMN suppression patterns during an n-back WM task in first-episode schizophrenia patients with and without cognitive deficits and the matched health controls. While the schizophrenia patients with preserved cognitive function suppressed the DMN as efficiently as the health controls did, the patients with impaired cognitive function showed significantly reduced DMN suppression in the mPFC and PCC. The correlation analyses showed that the greater DMN suppression was in the PCC node, the better WM performance was in both the HC and patient groups. Our findings lend further support to the notion that DMN dysfunction is involved in the neuro-pathological mechanism of cognitive impairment, specifically working memory deficit, in schizophrenia. This finding that reduction of DMN suppression in schizophrenia patients depends on the extent of cognitive impairment may provide a possible explanation for the inconsistent evidence on DMN suppression in schizophrenia^4^,^15^,^22^,^23^,^24^. Moreover, previous studies have demonstrated that the current pharmacological treatment cannot efficiently improve the cognitive deficits in schizophrenia^50^,^51^, our findings on the specific role of DMN suppression in cognitive deficits may help identify new drug targets for this severe mental disorder.

Efficient DMN suppression during externally goal-directed cognitive tasks has been suggested as essential for adaptive disengagement from distracting internal information processing^6^. This notion has been supported by the observation that the reduced DMN suppression is associated with attentional lapses^21^ and mind wandering^52^ in healthy subjects, and attention impairments in patients with traumatic brain injury^53^. Specially, our recent work has provided direct evidence showing a significant correlation between inefficient DMN suppression and attention disorder in schizophrenia^54^. This notion may be also consistent with Blueler’s proposal that the autistic thinking, one of the basic symptoms of schizophrenia, is characterized by the preponderance of inner life with an active turning-away from the external world^55^. Combining the proposed roles of DMN suppression, it is possible that the hyperactivity of DMN during WM task in schizophrenia patients reflects an inability to reallocate the attentional resources away from introspectively oriented mental activity and toward the demands posed by an external stimulus when performing cognitive tasks^8^,^21^,^48^. Thus, impaired DMN suppression may keep the schizophrenia patients consumed by introspectively oriented mental activity, leading to their inability to focus on the ongoing tasks^56^,^57^.

In other words, if the DMN is suppressed efficiently, individuals can flexibly reallocate their attention to focus on the external stimulus. The reduced DMN suppression observed in the SZ-Imp patients, but not in the SZ-Pre patients, suggests that the failure of DMN suppression is a potential biomarker for cognitive deficits of schizophrenia, thereby providing a possible neural basis for the varying cognitive performance in schizophrenia patients (DSM-IV)^26^. Notably, all patients in the present study were at the early-stage of schizophrenia and would not have been experienced potential cognitive declines from the secondary effects of chronic illness, such as prolonged social isolation and medication. Thus, the inefficient DMN suppression found in the SZ-Imp patients is more likely biologically driven. Moreover, the higher PCC activity in the SZ-Imp patients compared to the SZ-Pre patients also supports this role of DMN suppression. Although this between-group difference in PCC activity did not survive after FWE correction, the effect size (Cohen’s *d* = 1.39) was large enough to support the significance of this finding (uncorrected p < 0.001) in our data. Future studies with a larger sample are needed to replicate this finding.

Consistent with previous studies^5^,^9^, our correlation analyses showed a significant association between the DMN suppression and the cognitive performance in both HC and patient groups. This finding lends further support to the pathological involvement of DMN dysfunction in schizophrenia cognitive deficits. However, future longitudinal study is needed to determine the causal relationship between inefficient DMN suppression and cognitive deficits in schizophrenia. Moreover, this study found no correlation between the disrupted DMN suppression and the SAPS/SANS total score, age at illness onset, or duration of illness in the two patient groups, implying that DMN disruption during cognitive tasks may only relate to cognitive functioning in early-stage of schizophrenia. Furthermore, since the clinical and demographical variables between these two patient groups were well matched, higher PCC activity in the SZ-Imp group compared to the SZ-Pre group supports the notion that different DMN suppression patterns at the neurology level between the two patient groups account for their cognitive function differences at the behavior level^5^.

Interestingly, our correlation analysis identified a positive correlation between antipsychotic dosage and PCC activity in the patients. There are two possible explanations for this finding. One possibility is that the current pharmacological treatment has a deteriorating effect on DMN function in schizophrenia. This observation is consistent with the clinical phenomenon that the cognitive deficits of schizophrenia patients appear to be persistent and often resistant to antipsychotic drugs^58^. Another possibility is that the patients on higher dosage of antipsychotics may have a more severe illness, or be biologically distinct from those on smaller dosage. If so, it is probable that the patients on higher dosage of antipsychotics may present higher PCC activity, which has been consistently observed to be associated with more severe WM deficits in this study and previous researches^5^,^59^. However, our study design, which matched the medication dosage between two patient groups, prevented us from exploring this possibility further. Nevertheless, the effect of antipsychotics on the DMN suppression in schizophrenia calls for future longitudinal study on the drug-naïve patients.

Finally, although the effect sizes were large enough to support the significance of our findings in group comparisons of task-related brain activities, this study may be underpowered by the relatively small sample in the behavioral correlation analyses. For example, we detected a significant relationship between PCC activity and Target-accuracy of 2-back task only in patients, which contrasted with previous studies showing this significant correlations in both schizophrenia patients and healthy subjects^8^,^15^. Interestingly, using a Fisher’s r to z transform, our further analysis found no significant differences in the relationship of DMN suppression with WM performance between patients and healthy controls, suggesting that our correlation findings may be underpowered by the relatively small sample. Thus, future studies are needed to replicate these findings in a larger sample.

### Conclusions

This study provides the first direct evidence that the reduced DMN suppression during cognitive task exists only in first-episode schizophrenia patients with impaired cognitive function. Along with the association between the reduction of DMN suppression and poor working memory performance observed in the present study, our findings suggest a specific role of inefficient DMN suppression in the impaired cognitive processing in first-episode schizophrenia. DMN suppression may therefore be a neural target for the development of new medication in schizophrenia.

## Additional Information

**How to cite this article**: Zhou, L. *et al.* Inefficient DMN Suppression in Schizophrenia Patients with Impaired Cognitive Function but not Patients with Preserved Cognitive Function. *Sci. Rep.* **6**, 21657; doi: 10.1038/srep21657 (2016).

## Supplementary Material

Supplementary Information

## Figures and Tables

**Table 1:** Demographics, clinical characteristics and task performance of participants.

Demographics/clinical characteristics		Patients	
	Healthy participants n = 21 (10M:11F)	Preserved WM^a^ n = 19 (8M:11F)	Impaired WM^a^ n = 16 (11M:5F)
	Mean ± SD	Mean ± SD	Mean ± SD
Age (years)	22.38 ± 3.94	24.84 ± 6.68	22.63 ± 6.71
Education (years)	13.33 ± 1.83	12.63 ± 2.11	12.19 ± 2.69
WAIS-Digital	−	70.63 ± 16.11	59.25 ± 12.00
WAIS-Information	−	18.82 ± 4.98	15.72 ± 4.03
Age at illness onset (years)	−	24.11 ± 6.63	22.94 ± 6.63
Duration of illness (months)	−	8.25 ± 5.69	8.38 ± 4.24
CPZ equivalents (mg)	−	234.22 ± 133.99	319.79 ± 287.02
SAPS total	−	19.95 ± 8.53	14.19 ± 11.98
SANS total	−	24.05 ± 24.50	38.44 ± 23.44
WM Performances	Mean ± SD	Mean ± SD	Mean ± SD
2-back target accuracy	79.05 ± 13.07	75.74 ± 14.78	42.69 ± 18.30
2-back non-target accuracy	90.14 ± 7.30	90.58 ± 8.39	66.88 ± 37.90
2-back target RT	619.64 ± 125.26	689.87 ± 121.94	729.34 ± 205.07
2-back non-target RT	631.56 ± 121.09	695.30 ± 128.38	733.02 ± 160.13

*Note:*

^a^Defined by performance on the 2-back memory task; WM, working memory; M, male; F, female; SD, standard deviation; WAIS-Digital, the Digit Symbol Subscale of Wechsler Adult Intelligence Scale; WAIS-Information, the information subscale of Wechsler Adult Intelligence Scale; CPZ, chlorpromazine; SAPS, Scale for Assessment of Positive Symptoms; SANS, Scale for the Assessment of Negative Symptoms, RT, response time.
