# Supplementary material for: Inefficient DMN Suppression in Schizophrenia Patients with Impaired Cognitive Function but not Patients with Preserved Cognitive Function
Source: Sci Rep. 2016 Feb 17;6:21657. doi: 10.1038/srep21657 (PMC4756363; doi:10.1038/srep21657)
Supplement: Supplementary Information [file srep21657-s1.doc]

**Supplementary Materials for**

**Inefficient DMN Suppression in Schizophrenia Patients with Impaired Cognitive Function but not Patients with Preserved Cognitive Function**

**Li Zhou^1,7^, Weidan Pu^2,7*^, Jingjuan Wang^3^, Haihong Liu^4^, Guowei Wu^1,7^, Chang Liu^1,7^,** **Tumbwene** **E.** **Mwansisya^1,5^, Haojuan Tao^1,7^, Xudong Chen^1,7^, Xiaojun Huang^1,7^, Dongsheng Lv^1,7^, Zhimin Xue^1,7^, and Baoci Shan^3,6^, Zhening Liu^1,7,8^**

* Correspondence author: e-mail: [pulv1128@126.com](mailto:pulv1128@126.com).

Table S1

|  | **Cluster size** (voxels) | **Side** | | **MNI coordinates** | | **peak-level**  T value | |
| --- | --- | --- | --- | --- | --- | --- | --- |
|  |  |  |  | X Y Z | |  |  |
| **SZ-Imp > HC** |  | | | | | | |
| Temporal Pole | 127 | Left | | - 39 12 - 36 | | | 4.87 |
|  | 17 | Right | | 33 12 - 39 | | | 4.82 |
| Medial prefrontal cortex | 84 | Left | | - 6 54 9 | | | 4.65 |
| Posterior cingulate cortex | 47 | Left | | 0 - 54 24 | | | 4.05 |
| Para-Hippocamp | 23 | Right | | 24 - 6 - 24 | | | 4.68 |
| Angular gryus | 11 | Left | | - 48 - 66 33 | | | 3.64 |
| Anterior insula | 67 | Left | | - 36 6 0 | | | 4.16 |
| Anterior cingulate cortex | 13 | Left | | 0 9 33 | | | 3.76 |
| Caudate | 20 | Left | | - 3 9 - 9 | | | 3.72 |
| **SZ-Pre > HC** |  | | | | | | |
| Anterior insula | 30 | | Right | | 42 - 5 6 | | 4.27 |

Differences of Whole Brain Activities among Three Groups (p _uncorrected_ <0.001, cluster>10)

*Note:* SZ-Imp, schizophrenia patients with impaired cognitive function; SZ-Pre, schizophrenia patients with preserved cognitive function; HC, healthy controls.

Table S2

Head Movement Differences among Three Groups.

|  | **HC** | **Patients** |  | ***F*** | ***p*** |
| --- | --- | --- | --- | --- | --- |
| Head motion parameters |  | **SZ-Pre** | **SZ-Imp** |  |  |
|  | Mean ± SD | Mean ± SD | Mean ± SD |  |  |
| Translation (mm)**^a^** |  |  |  |  |  |
| X | 0.0881±0.0437 | 0.0708±0.0308 | 0.0830±0.0572 | 0.788 | 0.460 |
| Y | 0.1067±0.0929 | 0.1693±0.1780 | 0.1411±0.2458 | 0.633 | 0.535 |
| Z | 0.1074±0.0944 | 0.1087±0.0523 | 0.1142±0.0617 | 0.042 | 0.959 |
| Rotation (**°**) **^a^** |  |  |  |  |  |
| X | 0.0021±0.0014 | 0.0023±0.0013 | 0.0023±0.0009 | 0.155 | 0.856 |
| Y | 0.0015±0.0008 | 0.0012±0.0006 | 0.0012±0.0007 | 1.054 | 0.356 |
| Z | 0.0017±0.0015 | 0.0015±0.0010 | 0.0021±0.0028 | 0.508 | 0.604 |
| Translation (mm)**^b^** |  |  |  |  |  |
| X | 0.0180±0.0827 | -0.0064±0.0657 | -0.0100±0.0894 | 0.712 | 0.495 |
| Y | -0.0618±0.1186 | -0.1256±0.2095 | -0.1109±0.2568 | 0.573 | 0.567 |
| Z | 0.0121±0.1308 | -0.0143±0.1090 | 0.0165±0.1176 | 0.354 | 0.703 |
| Rotation (**°**) **^b^** |  |  |  |  |  |
| X | -0.0003±0.0023 | 0.0004±0.0024 | -0.0001±0.0022 | 0.478 | 0.623 |
| Y | -0.0002±0.0016 | 0.0003±0.0011 | -0.0005±0.0011 | 1.591 | 0.213 |
| Z | -0.0003±0.0021 | 0.0000±0.0017 | -0.0012±0.0032 | 1.168 | 0.319 |

*Note:* **^a^** Calculated with the Absolute value; **^b^** Calculated with the Raw value; HC, healthy controls; SZ-Pre, schizophrenia patients with preserved cognitive function; SZ-Imp, schizophrenia patients with impaired cognitive function; SD, standard deviation.
